# A thioacidolysis method tailored for higher‐throughput quantitative analysis of lignin monomers

**DOI:** 10.1002/biot.201600266

**Published:** 2016-09-14

**Authors:** Anne E. Harman‐Ware, Cliff Foster, Renee M. Happs, Crissa Doeppke, Kristoffer Meunier, Jackson Gehan, Fengxia Yue, Fachuang Lu, Mark F. Davis

**Affiliations:** ^1^ Bioenergy Science CenterGoldenCOUSA; ^2^ National Bioenergy Center, National Renewable Energy LaboratoryGoldenCOUSA; ^3^ Great Lakes BioEnergy Research CenterMichigan State UniversityEast LansingMIUSA; ^4^ Wisconsin Bioenergy InitiativeUniversity of WisconsinMadisonWIUSA

**Keywords:** Cell wall structure, Lignin, S/G ratio, Thioacidolysis

## Abstract

Thioacidolysis is a method used to measure the relative content of lignin monomers bound by β‐O‐4 linkages. Current thioacidolysis methods are low‐throughput as they require tedious steps for reaction product concentration prior to analysis using standard GC methods. A quantitative thioacidolysis method that is accessible with general laboratory equipment and uses a non‐chlorinated organic solvent and is tailored for higher‐throughput analysis is reported. The method utilizes lignin arylglycerol monomer standards for calibration, requires 1–2 mg of biomass per assay and has been quantified using fast‐GC techniques including a Low Thermal Mass Modular Accelerated Column Heater (LTM MACH). Cumbersome steps, including standard purification, sample concentrating and drying have been eliminated to help aid in consecutive day‐to‐day analyses needed to sustain a high sample throughput for large screening experiments without the loss of quantitation accuracy. The method reported in this manuscript has been quantitatively validated against a commonly used thioacidolysis method and across two different research sites with three common biomass varieties to represent hardwoods, softwoods, and grasses.

**Abbreviations**
BESC: BioEnergy Science Center
BPE: bisphenol‐E
DOE: Department of Energy
GC/MS: gas chromatography mass spectrometry
GLBRC: Great Lakes BioEnergy Research Center
LTM MACH GC/FID: low thermal mass modular accelerated column heater gas chromatography flame ionization detector
NREL: National Renewable Energy Laboratory
SG: switchgrass
S/G: ratio of syringyl monomer to coniferyl monomer within lignin
SIM: selective ion monitoring

## 1 Introduction

Conversion of biomass to renewable fuels and chemicals is an important aspect of renewable energy and materials research. Current efforts are focused on improving bioconversion technologies by reducing lignocellulosic biomass recalcitrance, leading to a reduction in biofuel production costs and competitive biomass‐derived products in the chemical and fuel markets. Methods used to reduce biomass recalcitrance have involved altering the structure and composition of the lignin within the biomass cell walls 1, 2, 3, 4, 5, 6. Lignin is an irregular biopolymer constructed from three phenylpropane units; *p*‐hydroxyphenyl or coumaryl (H), coniferyl (G), and syringyl (S), by various types of linkages. Understanding lignin structure and composition is essential for developing efficient methods to obtain renewable chemicals and materials from biomass. Recent work has shown that both lowering lignin content and altering the ratio of the monomer units can reduce biomass recalcitrance 1, 2, 4, 5. For example, hardwood lignins with higher S/G ratios degrade faster for certain conversion processes and increases in S/G ratios have been linked to increases in delignification rates 7. Increases in lignin solubility and pulping efficiency have also been linked to a higher content of S units in lignin 8.

**Figure 1:**
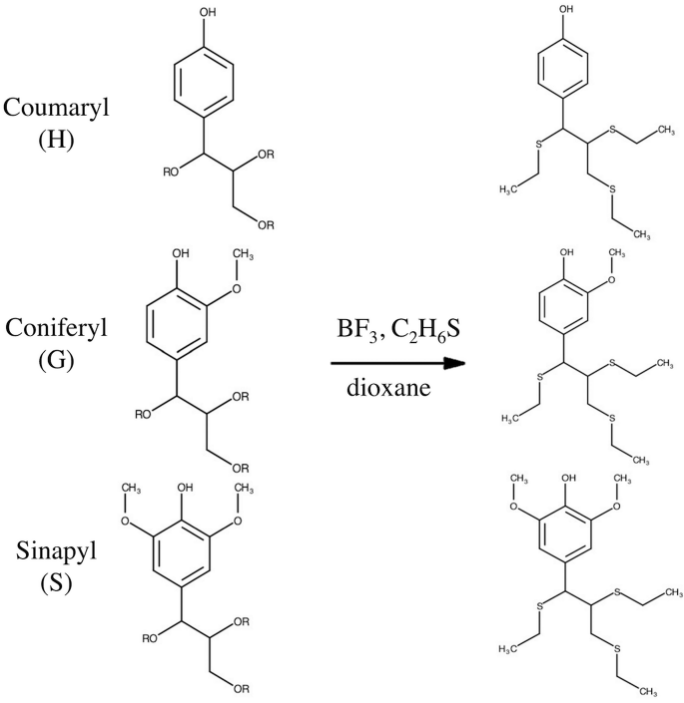
Thioacidolysis converts arylglycerol lignin monomers bound by β‐*O*‐4 linkages to corresponding thioethylated monomers.

Various types of chemical degradation techniques, such as thioacidolysis, nitrobenzene oxidation and derivatization followed by reductive cleavage (DFRC) methods, are capable of analyzing lignin structure and content 9, 10. Thioacidolysis of lignin, the reaction scheme in Fig. 1, is often preferred due to its ability to cleave ether linkages and efficiently release monomers 11. Thioacidolysis relies only on the cleavage of β‐*O*‐4 ether linkages to generate thioethylated H, G and S monomers 12, 13. Monomer products from thioacidolysis are analyzed by GC/MS and are typically quantified relative to an internal standard when authentic standards are not available. There are many chemicals and steps incorporated in a thioacidolysis reaction and typical methods are low throughput and labor intensive, requiring sample concentration and numerous postreaction workup steps followed by GC analysis using methods requiring at least 30 min/sample. Recently, the original thioacidolysis methodology has undergone some modifications aimed at high throughput analysis and improved quantitation using standards on GC/MS 14, 15, 16, 17.

The results presented describe a quantitative thioacidolysis technique tailored for high‐throughput analysis for screening large quantities of biomass. The rapid method eliminates laborious steps such as collection and purification of arylglycerol standard thioethylated products to generate calibration curves. Additionally, the method is microscale, does not utilize chlorinated solvents and eliminates steps used to concentrate the products prior to analysis utilizing previous improvements reported in Foster et al. 17. Further reductions in analysis time were accomplished by analyzing the thioethylated reaction products utilizing a low thermal mass modular accelerated column heater equipped gas chromatography instrument (LTM MACH GC/FID). The thioacidolysis method we report here was validated across two different laboratories and compared to a commonly used method in literature 15.

## 2 Materials and methods

### 2.1 Preparation of lignin monomer calibration standards

Syringyl, coniferyl and coumaryl arylglycerol monomers were synthesized according to the procedure reported in Yue et al. 14. Monomers were purified and stock solutions of 10 mg/mL of each monomer in dioxane (DriSolv, Fisher) were mixed and diluted in dioxane to generate standards with a range of S, G and H abundances (S/G/H = 1/1/0.25). Known volumes of standards were added to 1/2‐dram vials and the dioxane was gently evaporated using nitrogen to generate standards for thioacidolysis reactions ranging from 0.125 to 600 μg of each of the added monomers in the vials. 1000 μL of the thioacidolysis reaction mixture was added to each vial where the reaction and product work‐up was performed using the same method as described for biomass samples (Section 2.2). Calibration lines were generated by plotting the concentration ratio of each arylglycerol monomer with internal standard vs. the area ratio of the corresponding thioethylated products with internal standard to extrapolate the thioethylated product recovery from biomass samples.

### 2.2 High‐throughput thioacidolysis method

Prior to thioacidolysis, biomass samples were milled to 60‐mesh, extracted with ethanol and cryomilled. 2 mg of extracted biomass was weighed in to a 2 mL screw‐cap vial with a Teflon‐lined cap. Thioacidolysis reagent consisted of 2.5% boron trifluoride diethyl etherate (>47.5% BF_3_, Sigma Aldrich) 10% ethanethiol (97%, Alfa Aesar) and 87.5% dioxane by volume and contained bisphenol‐E as a surrogate internal standard (reagent grade, TCI Chemical) at a concentration of 0.05 mg/mL. 1000 μL of the thioacidolysis reagent was added to the vial containing biomass, purged with nitrogen, capped and heated to 100°C for 4 h. The reaction was quenched by cooling on ice for 5 min. The vial was then vortexed and the insoluble residue was allowed to settle prior to transferring 400 μL to a culture tube. The reaction was neutralized by adding 250 μL of 1 M aqueous sodium bicarbonate and then acidified with the addition of 2 N HCl sufficient to bring the pH down to 1 (100–130 μL). 1000 μL of water was added to the culture tube followed by 500 μL of ethyl acetate (99.9%, Fisher) and vortexed to ensure mixing. 2000 μL of water was added to the culture tube to increase the volume for pipetting and the tube was covered and allowed to sit for 10 min. 100 μL of the organic layer was then transferred to a GC vial (with insert). 10 μL of pyridine, 50 μL of bis(trimethylsilyl) acetamide (Sigma Aldrich) and optional 10 μL of 0.5 mg/mL tetracosane (additional internal standard) or ethyl acetate were added to the GC vial and allowed to sit for 2 h at room temperature prior to GC analysis. Silanized glassware should be used to ensure inertness and a fresh set of arylglycerol calibration standards should be run with each sample set.

### 2.3 Thioacidolysis product analysis

High‐throughput GC analysis of less than 5 min/sample is possible with the use of a Low Thermal Mass Modular Accelerated Column Heater (LTM‐MACH). An Agilent 7890B LTM Series II was used with a 10 m × 100 μm × 0.10 μm DB‐5 column coupled to the inlet and detector by 0.5 m of fused silica deactivated transfer lines. The inlet, main GC oven and FID were held at 250°C with a column flow of 0.6 mL/min. The LTM column tempera‐ture program began at 130°C for 2 min and was ramped at 150°C/min to 325°C. GC/MS analysis of the thio ethylated products can be performed using a 15 or 30 m × 250 μm × 0.25 μm DB‐5 column with or without a guard column (i.e. Phenomenex Zebron 10 m × 0.25 mm) with an oven program of 130°C to 300°C at 5°C/min and hold at the final temperature for 5 min. A 15 m GC column was used with a guard column at one laboratory and the 30 m column was used at the other; these parameters do not affect the analysis of the products when each lab references its own calibration standards. The inlet was held at 270°C and used a splitless single tapered inert liner and the transfer line to the MS was held at 280°C. The mass spectrometer source was 70 eV and run in Selective Ion Monitoring (SIM) mode for the following ions: 299 (thioethylated syringyl monomer TMS), 269 (thioethylated coniferyl monomer TMS), 239 (thioethylated coumaryl monomer TMS), 343 (bisphenol‐E internal standard). m/z 293 was also monitored to detect the presence of coumaric acid and m/z 338 was monitored for ferulic acid. The GC/MS oven ramp may also be increased for higher‐throughput (Lab 1) using the following temperature program: start 160°C, 2 min hold to 250°C at 25°C/min with a 4 min hold (~10 min/sample). Thioacidolysis products that have undergone work‐up for GC analysis may sit at room temperature for 24 h prior to injection, however, after piercing the vials, degradation of the silylated monomers was observed. Prior to each analysis consisting of a fresh set of calibration standards and reacted material, a new septum, GC liner and gold seal were installed and the column was trimmed. Reactive sites within unclean GC parts were found to influence product recoveries.

### 2.4 Validation of high‐throughput thioacidolysis method by cross‐laboratory tests and by comparison to another method

Different types of biomass with a range of S/G ratios were selected for analysis using the reported thioacidolysis method. Poplar (hybrid clone NE‐19 *Populus nigra charkowiensis × P. Nigra caudina*) provided by the Great Lakes BioEnergy Research Center (Laboratory 1) was used as a hardwood, high S/G ratio feedstock. NIST Monterey pine (*Pinus radiata*) was used as a softwood, low S/G ratio feedstock, and switchgrass (*Panicum virgatum*) provided by the Bioenergy Science Center at the National Renewable Energy Laboratory (Laboratory 2) was used as an herbaceous feedstock. Each sample underwent thioacidolysis at both Laboratory 1 and Laboratory 2. The method was developed for analysis of lignin present in extracted, cryomilled biomass and not tested on lignins isolated from biomass (i.e. Klason or organosolv lignin) as their linkages may be altered upon isolation, yielding thioethylated products that may not be demonstrative of the native lignin structure.

A previously reported thioacidolysis method was employed for validation and comparison purposes 15. The traditional validation method incorporates a sodium sulfate drying step using a vacuum manifold along with a concentration step that utilized a vacufuge resulting in more labor and lower throughput. The validation method also used more chemicals, including dichloromethane (less favorable with respect to safety and the environment) as the organic extraction solvent. The same aryl‐glycerol calibration standards were used for testing this method. Klason lignin content was determined using the method outlined in the NREL laboratory analytical procedure 18.

## 3 Results and discussion

### 3.1 Calibration using arylglycerol monomer thioacidolysis products

The calibration of the GC/MS and LTM‐MACH for the analysis of the thioacidolysis products is based on the method reported by Yue et al. 14. Briefly, syringyl (S), coniferyl (G) and coumaryl (H) arylglycerol monomers underwent thioacidolysis to generate the same thioethylated monomers obtained from β‐*O*‐4 linkages present in lignin polymers. Using SIM mode to detect and quantitate the monomers is recommended due to the typically low H monomer concentrations obtained from biomass and potential coelution with other species. Response factors for each thioethylated analyte relative to the internal standard were not generated as the concentration of the thioethylated products was not known; deviating from the calibration method reported in Yue et al. 14. For our high‐throughput analysis, the concentration of the starting arylglycerol monomer relative to internal standard was used as the independent variable and the area of the corresponding thioethylated product relative to the internal standard area was used as the dependent variable for the construction of calibration lines. Using the concentration of the starting arylglycerol monomer eliminates the need for the laborious purification of the thioethylated products from the arylglycerol monomers that would need to be produced in substantial quantities if a large number of biomass samples are analyzed to identify genes or quantitative trait loci (QTLs) associated with lignin content and/or S/G ratios 19. As reported in Yue et al., the thioacidolysis reaction of the arylglycerol monomers is approximately 90% efficient and hence, the molar concentration response factors should be comparable 14. Therefore, the method reported here substantially increases the throughput, ease of execution and cost of a similar method by eliminating the standard purification step.

**Figure 2:**
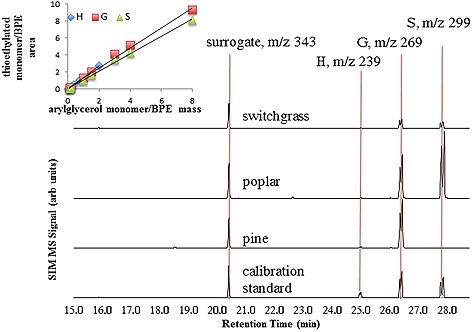
Selective ion chromatograms of thioacidolysis products from different types of biomass. Major ions used for quantitation of peaks are indicated, chromatograms are from Laboratory 2 GC/MS method. Calibration lines shown are obtained from single injection analysis of standards from a single reaction. Fresh calibrations should be referenced each time a reaction is performed.

The LTM‐MACH GC was capable of resolving the three monomers and the internal standard in less than five minutes. The use of the LTM‐MACH GC increases throughput of a typical GC method by at least 4‐fold due to the decreased run and column cooling time. Figure 2 shows SIM chromatograms (long GC/MS method) obtained from the thioethylated products of a calibration standard and the three biomass feedstocks. The calibration lines for each H, G and S monomer showed a high degree of correlation, *R* ^2 ^> 0.99 for all GC methods, indicating that the extent of reaction and derivatization in this method are not concentration dependent within the dynamic range. Additionally, this method is capable of detecting the analytes without having to concentrate the products in the post‐reaction workup. Coumaric and ferulic acids may be detectable using this method, but were not optimized for analysis and quantified in this report.

### 3.2 Product recoveries from high‐throughput thioacidolysis analysis of biomass

The yields of monomers and S/G ratios from all thioacidolysis reactions performed on biomass are summarized in Table 1. The standard deviation of the mass spectrometer area signal for a single poplar sample was found to be less than 2% for each ion monitored corresponding to the H, G and S monomers based on 10 injections. Total thioethylated monomer yields are reported as μmol/g biomass as opposed to μmol/g lignin since Klason lignin content may give different values depending on the laboratory, the purity and whether or not corrections for proteins were made. The ”traditional“ reaction that was performed for validation show similar trends to the method reported in this work while referencing the calibration standards and similar values for each biomass type as reported in the literature.

The LTM‐MACH GC/FID method produced similar results and trends as the other methods, although consistently lower for S, G and total monomers, which has been found using FID techniques previously 14. The H monomer recoveries were slightly higher for switchgrass and poplar based on the LTM‐MACH method which may be the result of other compounds generated from these particular feedstocks coeluting at the same retention time. H‐monomer recovery aside, the trend in product recoveries for each of the biomass types was the same for the LTM‐MACH method as the GC/MS methods. Overall, the LTM‐MACH method would provide rapid analysis for screening biomass samples for variation in S/G ratios and monomer recoveries (relative β‐*O*‐4 abundance). Lastly, the S/G ratio for each biomass type was determined to be the same value no matter the reaction method, laboratory or the GC method used. Coupled with the higher‐throughput thioacidolysis method described here, hundreds of biomass samples can be analyzed by thioacidolysis on a weekly basis.

**Table 1:** Monomer recoveries (μmol/g biomass) and S/G ratios of various types of biomass determined using thioacidolysis techniques.

Lab 1 – GCMS				Lab 2 – GCMS			Traditional Method ‐GCMS			LTM‐MACH GC/FID		
	SG	Pine	Poplar	SG	Pine	Poplar	SG	Pine	Poplar	SG	Pine	Poplar
Total Klason Lignin (wt%)	23.8	30.4	29.2	23.9	26.6	29.7	23.9	26.6	29.7	23.9	26.6	29.7
H	5.4 (±0.1)a)	7.9 (±0.9)	3.6 (±0.5)a)	12.0 (±0.2)a)	6.67 (±0.2)	5.5 (±0.0)a)	3.9 (±0.2)	6.3 (±0.3)	1.9 (±0.3)	15.6 (±0.6)	5.4 (±3.2)	25.9 (±2.3)
G	68.2 (±0.2)	198.6 (±32.4)	195.5 (±2.5)	68.7 (±1.43)	205.7 (±12.4)	200.7 (±12.3)	55.1 (±1.1)	215.1 (±9.1)	167.1 (±3.9)	55.5 (±3.6)	145.8 (±2.8)	183.2 (±14.4)
S	57.1 (±5.8)	5.0 (±0.1)a)	333.3 (±1.5)	52.8 (±0.9)	0.4 (±0.6)a)	344.2 (±20.7)	44.8 (±1.7)	6.7 (±0.0)	288.6 (±6.7)	43.5 (±2.0)	0.0 (±0.0)	297.9 (±14.3)
Total	130.7 (±5.5)	211.5 (±33.3)	532.4 (±4.0)	133.5 (±2.5)	212.7 (±13.2)	550.5 (±33.0)	103.8 (±3.0)	228.1 (±9.3)	457.6 (±10.7)	122.8 (±6.5)	151.4 (±7.8)	520.7 (±35.4)
S/G ratio	0.8 (±0.1)	0.0 (±0.0)	1.7 (±0.0)	0.8 (±0.0)	0.0 (±0.0)	1.7 (±0.0)	0.8 (±0.0)	0.0 (±0.0)	1.7 (±0.0)	0.8 (±0.0)	0.0 (±0.0)	1.6 (±0.1)

a) indicates likelihood of statistical difference (*p* < 0.05) between two laboratories analyses (*n* = 3). Samples were run in triplicate for each method comparing laboratories and duplicate for the traditional method and LTM‐MACH analysis.

The thioacidolysis results from each biomass type were typical using our higher‐throughput method in comparison to lower‐throughput methods reported elsewhere 11. Three different biomass types were tested in order to cover a range of lignin types, monomer recoveries and S/G ratios. Pine, like other conifers, is known to have little to no syringyl monomers within the lignin polymeric framework and our results agree with previous findings as a very low abundance of syringyl‐derived thioethylated products are detected (Table 1) 9, 10, 11, 14. The yield of G‐derived monomers from the pine was typical of reported yields from biomass of various types (accounting for lignin content) and in comparison to the ”traditional“ reaction based on Robinson et al. 10, 12, 14, 15. Based on *t*‐tests, both laboratories recovered statistically similar (unlikely to be different) amounts of H and G monomers from the thioacidolysis of the pine sample and also obtained similar S/G ratios. The low concentration of S monomer is likely the source of statistical differences in the S‐recoveries between the two laboratories.

Switchgrass total monomer yields were lower than the other two biomass types at both laboratories and were also observed in the traditional method. Low thioacidolysis yields for grasses have been reported in the literature, and have been attributed to the presence of condensed linkages (C‐C bonds) within the lignin polymer 9, 10, 11, 12, 15. The S/G ratio determined for the switchgrass was also typical based on findings reported by others 3, 9, 19, 20. The two laboratories recovered statistically similar S and G monomers and similar S/G ratios. Switchgrass is known to have H monomers present as coumarates within the lignin structure 21 and H monomers were detected, but in low abundance using this thioacidolysis method. The low concentration of H monomer is likely the source of statistical differences in the H‐recoveries between the two laboratories.

Poplar samples yielded the highest total amount of products. The high yields from the higher S/G ratio biomass are likely due to the higher S content of the lignin polymer leading to a higher occurrence of β‐*O*‐4 linkages. Both laboratories and both thioacidolysis methods recovered statistically similar amounts of G and S monomers from the poplar and had similar S/G ratios. H monomers were recovered in very small quantities and also were statistically different from the two laboratories. The S/G ratio of the poplar is similar to that reported by Yue et al. 14.

The total recoveries of monomers appear to be sensitive to the reagents used, moisture content of the biomass, and the cleanliness of the GC and could deviate in day‐to‐day analyses. Hence, dry reagents, dry biomass, silanized glassware, clean GC inlet parts and a trimmed column are necessary for accurate results and the consistent yields. Also, control samples and standards should be run in the same thioacidolysis reaction set as any experimental samples for comparisons and observations of experimental error.

## 4 Concluding remarks

A higher‐throughput and quantitative thioacidolysis method was applied to different types of biomass and the product recoveries were reported relative to arylglycerol standards that simultaneously underwent thioacidolysis. The products from the higher‐throughput thioacidolysis method were quantified using fast GC methods, particularly an LTM‐MACH GC/FID that used a method 4 times faster than a typical GC method. Thioethylated products and S/G ratios were similar in value and trends between two laboratories, across different GC methods and to those reported in the literature and for a previously reported thioacidolysis method for the types of biomass analyzed here. Collection and purification of the standard thioethylated products was not necessary to generate calibration curves resulting in higher‐throughput and lower labor costs than a similar method. Additionally, the method reported herein is microscale (2 mg), does not utilize chlorinated solvents or specialized equipment, and eliminates laborious steps necessary in similar methods to purify the standards, dry the reaction organic layer and concentrate the products. Overall, this thioacidolysis method is robust and capable of screening large populations of various types of lignocellulosic biomass for monomer composition at a higher‐throughput pace.
